# Endothelial cells dictate cardiac fuel source

**DOI:** 10.18632/aging.101825

**Published:** 2019-02-13

**Authors:** Jacqueline Taylor, Andreas Fischer

**Affiliations:** 1 Division Vascular Signaling and Cancer (A270), German Cancer Research Center (DKFZ), 69120 Heidelberg, Germany; 2 Department of Medicine I and Clinical Chemistry, University Hospital of Heidelberg, 69120 Heidelberg, Germany; 3 European Center for Angioscience, Medical Faculty Mannheim, Heidelberg University, 68167 Mannheim, Germany

**Keywords:** blood vessels, angiocrine, fatty acids, muscle, Notch signaling

Blood vessels can no longer be seen as just passive conduits for delivering blood. Endothelial cells lining blood vessels separate blood from surrounding parenchymal cells. This unique position allows endothelial cells to sense the presence of nutrients in blood plasma and to subsequently control their flux into tissue. Recent work demonstrates that the movement of metabolites across the endothelial barrier occurs in a highly regulated manner [1–3] and that dysfunction of this process may even contribute to the development of heart failure [1].

The organ-specific endothelium provides a rich set of membrane-bound and secreted factors (referred to as angiocrine factors), which coordinate organ development, tissue regeneration, the maintenance of stem cells and metabolism [4]. Therefore, endothelial dysfunction may not just be the consequence of metabolic diseases such as diabetes mellitus; instead, dysfunctional endothelial cells might be a primary instigator of subsequent organ damage.

Here, we discuss one such aspect, namely endothelial control of long chain fatty acid (LCFA) transport to myocytes and its implication for cardiac function. LCFAs are the most abundant energy source for cardiac muscle cells. Although still under debate, it is assumed that a significant amount of circulating LCFAs reach the cardiomyocytes after first being taken up by cardiac endothelial cells and subsequently being released again at the basal site. Their active transport across the continuous endothelium is facilitated by a receptor-mediated mechanism in which the scavenger cluster of differentiation-36 (CD36) is involved. Son et al. [2] have recently demonstrated that CD36 in the endothelium of heart and skeletal muscle is compulsory for adequate uptake of circulating fatty acids into muscle tissue. This work also showed that endothelial-mediated impairment of fatty acid uptake in muscle tissue is followed by increased glucose uptake and consumption [2], most likely to compensate the need for an energy source. But where are LCFAs then consumed or deposited? There is solid evidence that the liver clears excessive LCFAs, which can cause fatty liver disease upon accumulation [1,2]. This can be explained by the different architecture of blood vessels in liver where endothelial cells form a discontinuous sinusoidal endothelial layer, which provides large pores allowing nutrients to directly reach hepatocytes without any need for trans-endothelial transport [5].

To present knowledge, little is known as to how endothelial transport of LCFAs is actively regulated. Vascular endothelial growth factor B has been shown to regulate the transcription of genes of the fatty acid transport protein (FATP) family in addition to its role in inducing blood vessel expansion in the heart [6,7] Our group has reported that endothelial Notch signaling is a transcriptional regulator of several proteins, including CD36, which are needed for fatty acid uptake and transcytosis in endothelial cells (Figure 1) [1]. Genetic inhibition of canonical Notch signaling in endothelial cells of adult mice diminished the transcription of endothelial lipase, CD36 and fatty acid binding protein 4 (FABP4). It also led to increased expression of angiopoietin-like 4 (ANGPTL4), an inhibitor of lipoprotein lipase. As a consequence, the hydrolysis of triglycerides into free fatty acids as well as the uptake of radioactively-labeled LCFAs into heart and skeletal muscle was diminished [1]. Similar to the deletion of CD36 in the endothelium [2], inhibition of endothelial Notch signaling led to a metabolic shift to favor glucose oxidation as a fuel source.

**Figure 1:**
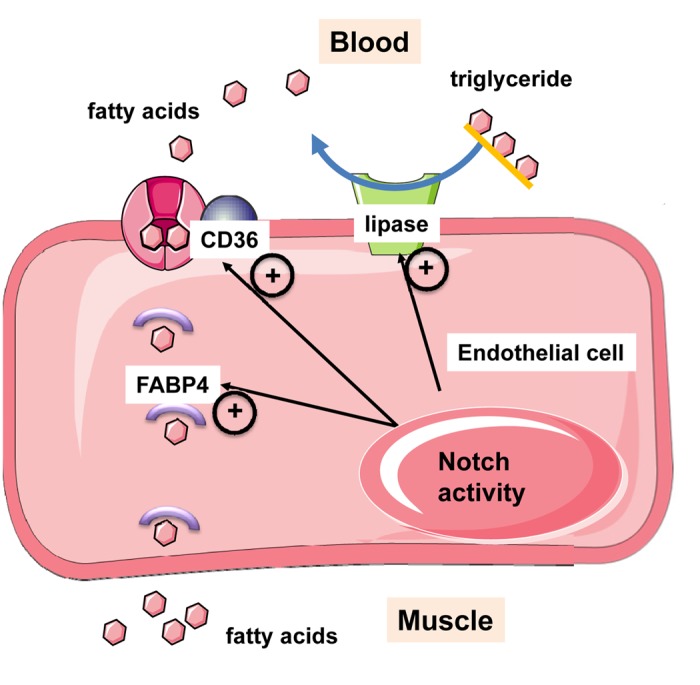
**Notch signaling in endothelial cells induces transcription of genes needed for trans-endothelial flux of fatty acids.** Endothelial Notch signaling induces expression of endothelial lipase for the hydrolysis of triglycerides into free fatty acids. Long chain fatty acids are taken up by fatty acid transporters and CD36, and shuttled through the cell by fatty acid binding protein-4 (FABP4). After release at the basal site, fatty acids can be taken up by myocytes.

What are the functional consequences of a metabolic shift from fatty acid to glucose consumption? Under normal conditions, heart, skeletal muscle and brown adipose tissue rely on fatty acids for energy in order to meet their high ATP generation needs. During aging, but also in several experimental models of heart failure, cardiomyocytes shift to an enhanced reliance on glucose metabolism. Our recent work indicates that such a metabolic shift – by interfering with endothelial LCFA transport to myocytes – can already accelerate heart failure in mice. We cannot entirely rule out that loss-of-Notch-induced changes in vascular morphology could contribute to cardiac dysfunction, however, feeding mice with a ketogenic diet strongly improved cardiac function [1], therefore indicating the importance of the metabolic fuel source as a driver for heart failure. Mechanistically, ketone bodies are transported by monocarboxylate transporters through the endothelium. Thereby, they can replace fatty acids as a sufficient fuel source for persistent β-oxidation even in the absence of fatty acid transporters. In addition, the benefit of a ketogenic diet in prolonging lifespan has also been shown in the context of age-associated heart failure in mice [8]. The molecular mechanisms behind these beneficial effects need to be studied more deeply, however, it is remarkable how a change in fuel source has such potent pathological consequences.

In summary, endothelial-mediated active transport of metabolites can govern the function of the supplied organ by regulating nutrient availability to parenchymal cells, and should be taken into account in future therapeutic interventions in tackling aging-related disorders.

## References

[1] Jabs M, et al. Circulation. 2018; 137:2592–608. 10.1161/CIRCULATIONAHA.117.029733 29353241

[2] Son NH, et al. J Clin Invest. 2018; 128:4329–42. 10.1172/JCI99315 30047927 PMC6159965

[3] Crewe C, et al. Cell. 2018; 175:695–708.e13. 10.1016/j.cell.2018.09.005 30293865 PMC6195477

[4] Rafii S, et al. Nature. 2016; 529:316–25. 10.1038/nature17040 26791722 PMC4878406

[5] Augustin HG, Koh GY. Science. 2017; 357. 10.1126/science.aal2379 28775214

[6] Hagberg CE, et al. Nature. 2010; 464:917–21. 10.1038/nature08945 20228789

[7] Kivelä R, et al. EMBO Mol Med. 2014; 6:307–21. 10.1002/emmm.201303147 24448490 PMC3958306

[8] Newman JC, et al. Cell Metab. 2017; 26:547–557.e8. 10.1016/j.cmet.2017.08.004 28877458 PMC5605815

